# Charge Variants of an Avastin Biosimilar Isolation, Characterization, *In Vitro* Properties and Pharmacokinetics in Rat

**DOI:** 10.1371/journal.pone.0151874

**Published:** 2016-03-17

**Authors:** Yan-Yan Zhao, Ning Wang, Wan-Hui Liu, Wen-Jie Tao, Li-Li Liu, Zhen-Duo Shen

**Affiliations:** 1 School of Pharmacy, Collaborative Innovation Center of Advanced Drug Delivery System and Biotech Drugs in Universities of Shandong, Key Laboratory of Molecular Pharmacology and Drug Evaluation (Yantai University), Ministry of Education, Yantai University, Yantai, 264005, P.R. China; 2 State Key Laboratory of Long-acting and Targeting Drug Delivery System, Luye Pharma Group Ltd., Yantai, 264005, P.R. China; Kermanshah University of Medical Sciences, ISLAMIC REPUBLIC OF IRAN

## Abstract

The similarity between a proposed biosimilar product and the reference product can be affected by many factors. This study is designed to examine whether any subtle difference in the distribution of the charge variants of an Avastin biosimilar can affect its *in vitro* potency and *in vivo* PK. Here, the acidic, basic and main peak fractions of a biosimilar product were isolated using high-performance cation-exchange chromatography and were subjected to various studies to compare their *in vitro* properties and *in vivo* PK profile. A serial of analytical methods, including size exclusion chromatography (SEC), imaged capillary isoelectric focusing (icIEF) capillary zone electrophoresis (CZE) and cation-exchange chromatography (CEX-HPLC) were also used to characterize the isolated charge variants. The kinetics constant was measured using a Biacore X100 system. The study indicates the biosimilar product has a high similarity with avastin in physicochemical properties. The potency in vitro and PK profile in rat of charge variants and biosimilar product are consistent with avastin.

## 1. Introduction

Monoclonal antibodies (mAbs) have become an important class of therapeutic proteins and the fastest growing class of therapeutic agents due to their advantages of being highly specific and relatively homogeneous [1–3]. As the patents of many original biologics expire, the development of biosimilar products with similar quality, safety and efficacy to the original biologics will improve the accessibility of biotherapeutic drugs to patients. Many regulatory agencies worldwide have already made guidelines to regulate the development of biosimilar products in their countries.

Despite their medical advantages, most biologics, especially mAbs, have high molecular weights and complicated structures, posing a challenge to the development of biosimilars. In addition, mAb products have heterogeneous variants due to a series of post-translational modifications that arise during cell culture, purification and storage. Such modifications may include oxidation, deamidation, amino acid substitution/deletion, differential glycosylation, glycation, isomerization, succinimide formation, N-terminal pyroglutamic acid formation, and C-terminal lysine clipping [4–6]. Some of these modifications can alter the charge distribution on the surface of the mAb and result in charge variants. It has been reported that the charge variants of recombinant mAbs show no substantial difference in the serum PK profile [7]. There are also literature reports suggesting that shifts of approximately one isoelectric point (pI) or more and charge variants resulting from chemical modification potentially affect the tissue distributions and pharmacokinetics (PK) profiles of mAbs [8–11]. Product consistency is an important factor that provides flexibility in manufacturing and supply management, and it is necessary to evaluate charge heterogeneity for the assurance of the quality and stability of mAb products.

Avastin is a recombinant humanized monoclonal IgG1 antibody developed by Roche that has become one of the best-selling drugs for cancer treatment worldwide. It inhibits the biological activities of vascular endothelial growth factor (VEGF) to block the blood supply of tumors and prevent the metastasis of cancer cells in the body [12]. Avastin combined with chemotherapeutics has shown a generally acceptable tolerability profile for patients with ovarian cancers, lung cancers, advanced cancers and predominant liver metastases. It enhances the effects of chemotherapy and significantly prolongs both progression-free survival and overall survival [13–15].

To demonstrate whether there are differences in the *in vitro* activity and PK profile among the charge variants, the acidic, basic and neutral variants (main peak) were prepared from a biosimilar product of Avastin by strong cation exchange chromatography [7, 16], and they were further characterized by various analytical techniques, such as weak cation-exchange chromatography (CEX-HPLC) to determine purity, capillary zone electrophoresis (CZE) to provide complementary information, size exclusion chromatography (SEC) to determine monomer percentage, Biacore X100 to determine kinetics constants [17–19] and imaged capillary isoelectric focusing (icIEF) to determine pI [20–26].

The activities of the isolated charge variants, biosimilar product and Avastin were determined using Human Umbilical Vein Endothelial Cells (HUVEC) [27–30]. The ability of all samples to inhibit the proliferation of a cultured cell line was measured. The pharmacokinetic studies were conducted in male Sprague-Dawley (SD) rats with single IV administration dosing using Avastin as a reference.

## 2. Materials and Methods

### Ethics Statement

All studies were conducted in accordance with the principles of Laboratory Animal Care (NIH publication no. 92–93, revised in 1985), and the ethical approval was granted by the institutional review board of the Yantai University. Written informed consent was obtained for all subjects. Protocols were designed to minimize pain and discomfort during the procedure and the animals were returned to their home cages after the study.

The Human Umbilical Vein Endothelial Cells (HUVEC) were purchased from Promocell. PromoCell is the original manufacturer of all primary, stem, and blood cells featured in their catalog and is committed to the highest ethical and legal standards. The tissue used by PromoCell for the isolation of human cell cultures is derived from donors who have signed an informed consent form (by the donor, an authorized agent, or a legal agent) that outlines the purpose of the donation and the procedure for processing the tissue in detail, and the procedure was approved by the IRB. They do not accept or use any tissue without prior signing of the consent documents.

### 2.1 Materials

Ultrapure 2-(N-morpholino) ethanesulfonic acid (MES) (>99.5% titration), sodium chloride, and sodium hydroxide were purchased from Sigma-Aldrich (St. Louis, MO, USA). The recombinant mAb was generated in the Antibody Technology Department of Luye Pharma Group. The protein was expressed in Chinese Hamster Ovary cells and purified by a three-step purification process including ProteinA, cation-exchange and anion-exchange chromatography. Avastin was purchased from Roche (Swiss). Carrier ampholytes with pH ranges of 3–10 and 8–10.5 were obtained from GE Healthcare. The pI markers were purchased from Protein Simple (Santa Clara, CA). Human Antibody Capture Kit was purchased from GE Healthcare Life Sciences and it was intended for use in capture of human or humanized IgG antibodies as ligands in biomolecular interaction analyses using Biacore X100 system. Bovine serum albumin (BSA) was obtained from Sigma (USA). The CM5 sensor chip (research grade) and amine coupling reagents (0.05 M N-hydroxysuccinimide [NHS], 0.2 M N-ethyl-N0-(3-diethylamino-propyl) carbodiimide [EDC], ethanolamine pH 8.5, HBS–EP buffer 0.01 M Hepes (pH 7.4) were obtained from GE. The sepharose high performance (SP-HP) column and MonoS^™^ 10/100 GL column were purchased from GE Health (Sweden). The expression plasmid pSVID5.ID.LLnspeV.xvegf36HC.LC encoding bevacizumab was introduced into Chinese hamster ovary parental cells CHO DP-12 by lipofection and cells were selected in the presence of increasing concentrations of methotrexate (MTX). Isolates were selected for secretion of active bevacizumab.

### 2.2 Preparation of charge variants

A GE AKTA AVANT chromatography system was used for all runs. The charge variants were prepared using an SP-HP column and a MonoS^™^10/100 GL column [31–32]. The acidic variant was first separated from the biosimilar product using the SP-HP column, and the elution buffer was 25 mM MES, pH 5.9 and 75 mM NaCl. The basic variant and main peak were then eluted by 2 M NaCl. After desalting, the basic variant and main peak were separated by the MonoS column. Elution buffer A was 25 mM MES, pH 5.9 and 50 mM NaCl, and buffer B was 25 mM MES, pH 5.9, and 500 mM NaCl. A generic linear gradient starting from 5–8% B gradient in 20 column volumes was used to separate the basic variant and main peak at a flow rate of 4 mL/min. The separation was monitored at 280 nm, and each fraction was injected into a weak cation exchange column to assess the purity.

The charge variant samples were concentrated and the buffer exchanged using an Amicon Ultra-15 (30,000 MWCO, from Millipore) ultrafiltration enrichment centrifuge tube. The final sample solution contained 30 mg/mL protein, 120 mM trehalose and 0.02% polysorbate 20. Samples were sterile-filtered using Sartolab Disposable Sterile Filter Systems and Bottle Top Filters (0.22 μm, Sartorius Stedim Biotech). The obtained samples were characterized using CEX, CZE, SEC, and icIEF. Protein concentrations were spectrophotometrically determined by absorbance at 280 nm using a NanoDrop 2000 (Thermo Scientific).

### 2.3 Analytical cation-exchange analysis (HPLC assay)

The purity of the isolated charge variants and starting material was determined by weak cation-exchange chromatography using a ProPac WCX-10 analytical column (4×250 mm, Dionex). Mobile phase A was 20 mM MES, 50 mM NaCl, pH 5.6, and mobile phase B was 20 mM MES, 240 mM NaCl, pH5.6. The initial concentration of phase B was 30%, and a linear gradient was run from 30% to 70% over 35 min followed by a linear increase from 70% to 100% over 0.1 min and a constant 100% flow from 35.1 to 40 min. The reference and charge variant samples were diluted with mobile phase A to approximately 0.25 mg/mL. The column was flushed and equilibrated with the initial running buffer until the baseline was stable. Peak areas were quantified relative to monobody, and protein was detected at 214 nm.

### 2.4 Analytical size-exclusion analysis (SEC)

The relative amount of monomer was determined by analytical SEC using a TSK-Gel column G3000 SWXL 7.8 mm×300 mm (Tosoh Biosep) at ambient temperature on an Agilent 1200 HPLC (Agilent Technologies) dual wavelength absorbance detector at 280 nm. The sample was diluted to 0.5 mg/mL using the mobile phase (0.35 mol·L^−1^ sodium phosphate, 0.35 mol·L^−1^ sodium chloride) and injected after filtration using 0.45 μm GHP Acrodisc minispike syringe filter (Waters). The chromatograms were analyzed by integrating the area under each elution peak and recorded as percentages of aggregate and monomer.

### 2.5 Imaged capillary isoelectric focusing analysis (icIEF)

The icIEF experiments were performed on the PA 800 Protein Characterization System (Beckman Coulter, CA), and the separation consisted of two steps, i.e., focusing and mobilization [33–35]. The solution used in the experiment included 4% V/V carrier ampholytes (pH 3–10 and 8–10.5 ampholytes with a ratio of 1:1), 0.35% MC W/V, 0.5% V/V pI markers 7.40 and 8.79, and a final sample concentration of 0.5 mg/mL. To focus the sample, a potential of 1000 volts was first introduced for 1 min, followed by a potential of 2000 volts for 1 min, and finally a potential of 3000 volts was applied for 6 min. During the focusing step, the sample migrates towards the point at which its net charge is zero. At this point, the sample is focused in a tight zone, and the migration stops. Detection was performed at 280 nm.

### 2.6 Capillary zone electrophoresis assay (CZE)

All samples were analyzed on a Beckman PA 8000 capillary electrophoresis system equipped with a 50 μm diameter uncoated fused-silica capillary. The separation proceeds according to differences in the analyte electrophoretic mobilities depending on their charge-to-size ratio. Before performing the experiment, the capillary was first preconditioned by flushing deionized distilled water, 0.1 M HCl, and CZE running buffer (500 mM Aminocaproic acid) for 9 min at a pressure of 50 psi separately and then applying a normal polarity voltage of 30 kV for 10 min. Conditioning steps before each injection was performed by flushing the capillary with 0.1 M HCl and running buffer for 2 min at a pressure of 50 psi, respectively. Separation was performed at 30 kV in normal polarity for 10 min at 20°C after sample was injected for 10 s at 0.5 psi. Column storage for long term use: 0.1 M HCl in capillary with two ends dipped in water.

### 2.7 Affinity and kinetics assay

All binding and kinetics studies were performed on a Biacore X-100 instrument (GE Healthcare) by surface plasma resonance (SPR). The sensor biochip CM5 was activated using EDC/NHS at 10 μL/min for 7 minutes. Goat anti-human IgG Fc mAb was immobilized on two flow cells of the CM5 sensor chip at a concentration of 25μg/ml in 10 mM sodium acetate buffer (pH 5.0) for 7 min at 10 μL/min. HBS–EP was used as the running buffer and blank runs were performed with HBS–EP buffer (or an appropriate buffer for all other experiments). The sample was injected onto the senser chip with a flow rate of 10 μL/min. A series of antigen concentrations including 6.25 nM, 12.5 nM, 25 nM, 50 nM and 100 nM were injected consecutively, each with a contact time of 120 seconds and a dissociation time of 1800 seconds. The chip was regenerated using 3 M magnesium chloride. The datas were analyzed using the BiacoreX100 analytical and evaluation softwares (GE Healthcare). The dissociation rate constant (k_d_) and the association rate constant (k_a_) were calculated by a global fitting analysis. The first dissociation equilibrium constant (K_D1_) was determined from the ratio of the rate constants: K_D_ = k_d_ / k_a_.

### 2.8 Anti-proliferation assay

This particular cell-based assay was aimed at evaluating the potency of the isolated charge variants *in vitro* by measuring their ability to inhibit the proliferation of Human Umbilical Vein Endothelial Cells (HUVEC) expressing the receptor compared with the reference (Avastin). In brief, HUVEC cells were re-suspended in basal medium containing 2% fetal calf serum (FCS) without any growth factor. Approximately 1000 cells were seeded in each well of a collagen I-coated 96-well plate and incubated overnight. A series of diluted samples (20 μg/mL-0.005 μg/mL) and Avastin mixed with 30 ng/mL VEGF were added to the wells (100 μL per well). The plate was incubated for 72 h, and alarmablue was added to the plate at 20 μL per well. The absorbance of each well was measured on a fluorescence 96-well plate reader with an excitation wavelength of 560 nm and emission wavelength of 590 nm. The IC50 values of the samples and Avastin were calculated by a standard concentration curve, and the results were expressed as the relative activity (sample/Avastin).

### 2.9 Intravenous pharmacokinetic study data analysis in normal rats

#### Animal experiment

Sprague-Dawley male rats with a mean weight of 180–220 g were obtained from the Experimental Animal Center of Luye Pharma Group. The rats were allocated randomly into five groups (4 rats per group). The first group was administered the acidic variant, the second group was administered the basic variant, the third group was administered the main peak, the fourth group was administered the biosimilar product, and the fifth group was administered Avastin. All formulations were given at a dose of 10 mg/kg body weight *via* the caudal vein. Blood samples (approximately 0.5 mL) were collected from the canthus pre-dose and post-dose at 5, 10, 15 min; 1, 4, 8, 24 h; and 2, 3, 7, 10, 14, 17, 21, 24, 27, 31, 35, 42 days. Serum samples were prepared by centrifugation at 13000 r/min for 5 min at 4°C and stored at -70°C until analysis [36].

#### Determination of serum concentration

Serum concentration was determined using a validated Enzyme Linked Immunosorbent Assay (ELISA). First, 0.05 mg/mL of the antigen in 0.01 M phosphate buffer was added to 96-well plate polystyrene microtiter plate, which was then incubated at 37°C for 2 h. Each well was aspirated and washed with 250 μl of PBS Tween four times. The wells were then blocked with 200 μL of 1% bovine serum albumin/phosphate tween (BSA/PBST). After 15 seconds of agitation, the plate was incubated at 37°C for 1 h. The same washing procedure described above was performed before adding 100 μL of diluted sample, standards and controls. The plate was agitated for 15 seconds and incubated at 37°C for 1 h. After washing the plate, 100 μL of mouse anti-human IgG Fc conjugated to horseradish peroxidase was diluted 1:3000 in assay buffer and added to each well. The plate was incubated at 37°C for 1 h followed by another washing procedure. Then, 100 μL of a 1:1 mixture of tetramethylbenzidine (TMB) peroxidase substrate [Kirkegaard & Perry Labs (KPL) 50-76-0] and peroxidase solution B, 0.02% hydrogen peroxide (KPL 50-65-00), was then added to each well. After 30 minutes of incubation, the reaction was stopped by adding 100 μL of 1 M phosphoric acid, and the color changed from blue-green to yellow. The serum concentrations were determined using the SoftMax Pro V5 software package (five-parameter logistics regression; Molecular Devices).

## 3. Results and Discussion

### 3.1 Preparation and characterization of charge variants

A biosimilar product containing 15% acidic variant, 75% main peak and 10% basic variant was used for the preparation of the charge variants. The acidic variant fraction was obtained using a SP-HP column, and the basic variant fraction and main peak were obtained using a MonoS column. The MonoS column with a 10 μm particle size has higher resolution than SP-HP, which has 34 μm particle size, and the charge variants were obtained in sufficient amounts for the following studies [32]. Each individual charge variant and the starting material were buffer-exchanged into formulations of 120 mM trehalose and 0.02% polysorbate20, and the concentrations were adjusted to 30 mg/mL.

#### CEX-HPLC

**Fig 1:**
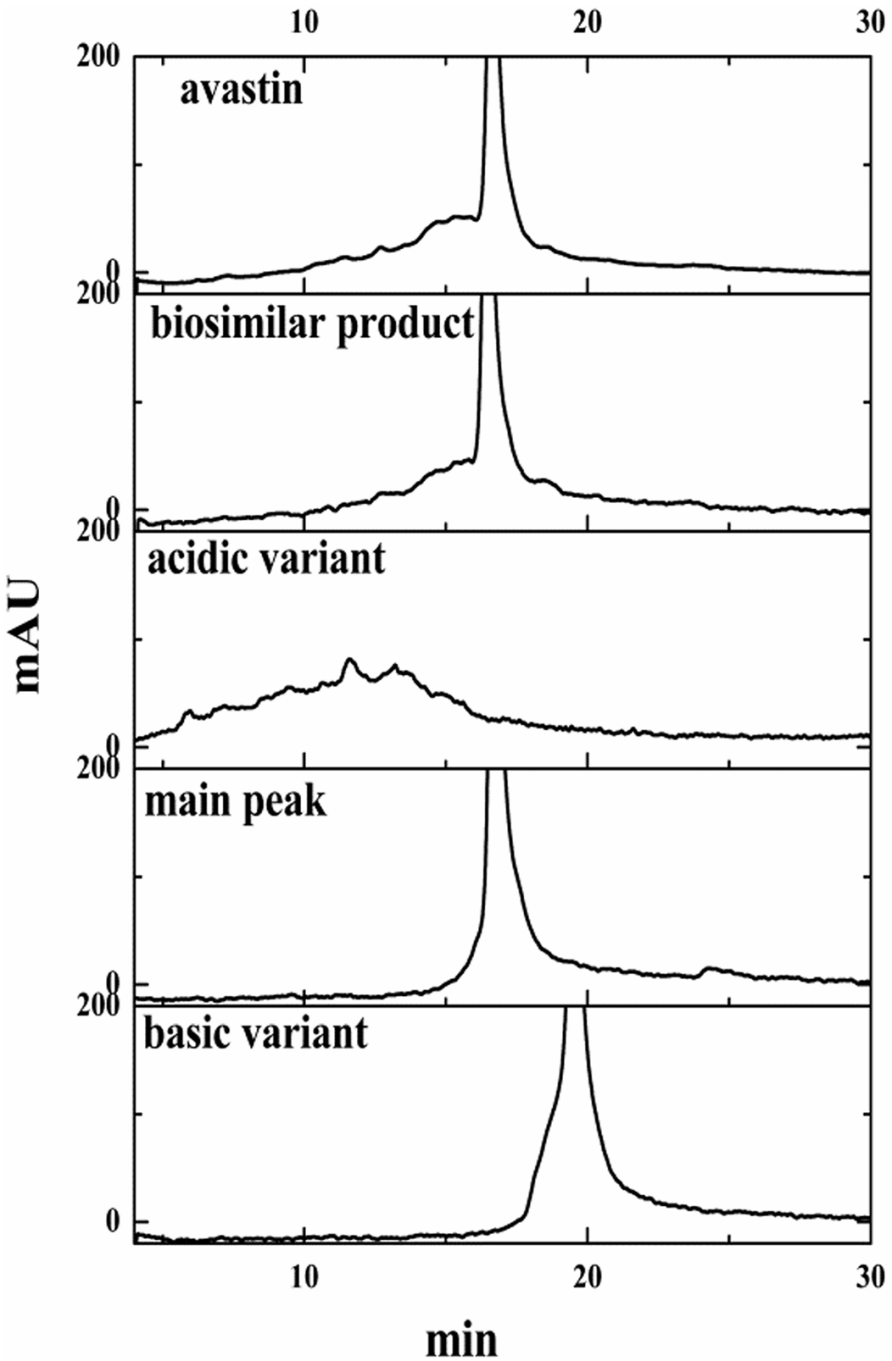
Chromatographic profiles obtained from a CEX-HPLC shown in expanded scale for the isolated charge variants, the biosimilar product and Avastin.

The purity of the charge variants, the biosimilar product and Avastin were determined by the weak cation-exchange method. The biosimilar product consisted of approximately 15% acidic variant, 10% basic variant and 75% main peak, respectively. The result was shown in Table 1. Moreover, the primary acidic variant, basic variant and main peaks were found in the acidic region, basic region and main region of the CEX chromatogram, and the purities of the respective charge variants were 95%, 90% and 91%. The CEX chromatograms are shown in Fig 1.

**Table 1:** Analytical results by the CEX-HPLC method for the biosimilar product and Avastin.

Test material	Acidic variant (%)	Basic variant (%)	Main peak (%)
biosimilar product	15	10	75
Avastin	15	11	74

#### CZE

**Fig 2:**
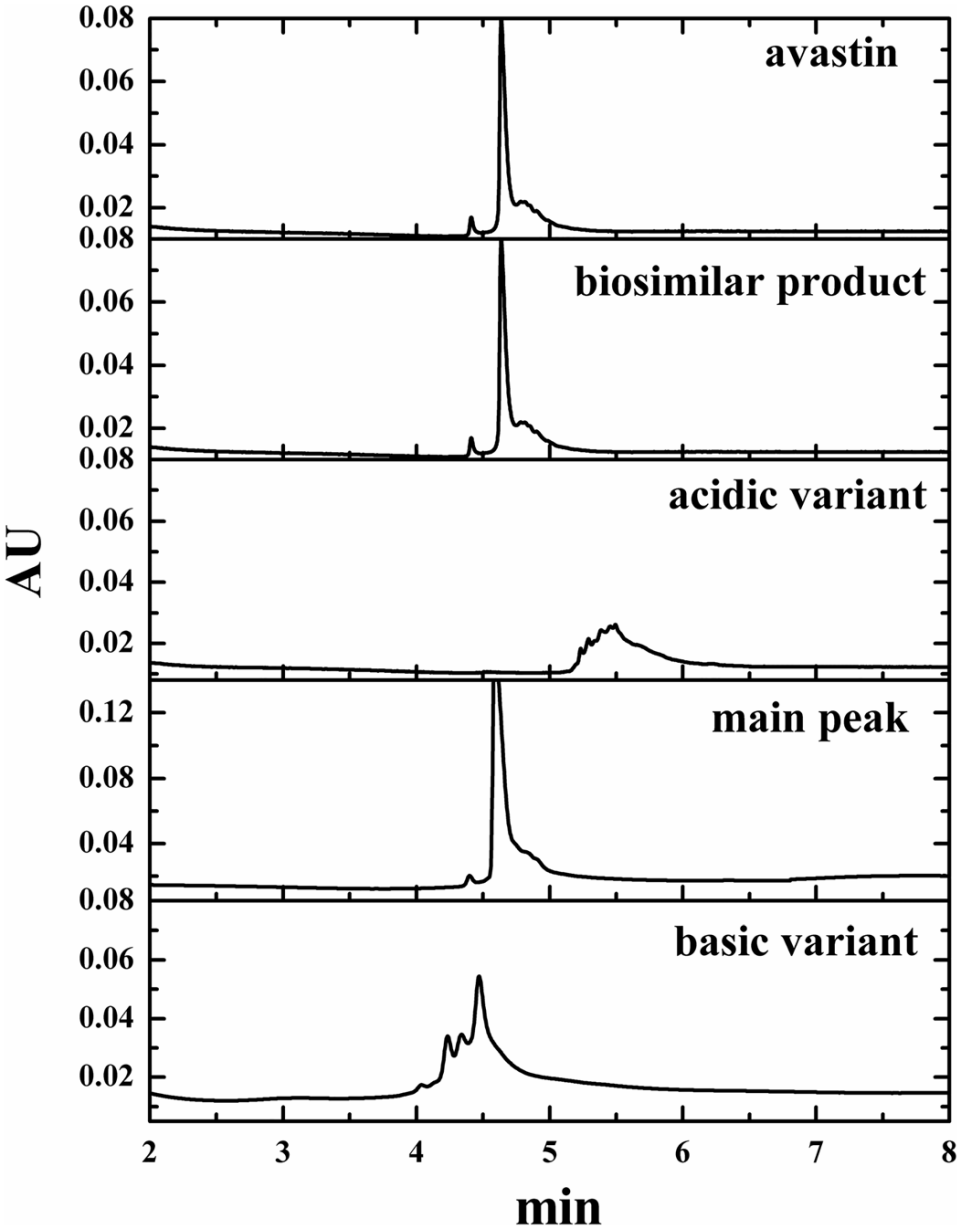
Charge variants of mAbs, biosimilar product and avastin profile obtained by CZE analysis.

This method is also a tool for analysis of charge heterogeneity of mAbs and provide complementary information. The molecular migrate with different speeds when subjected to an electric field, with the basic variants eluting first, followed by the main peak, and then the late eluting acidic variants [37–38]. The composition of the biosimilar product and avastin was consistent. Meanwhile, the purity of acidic variant, basic variant and main peak were 94%, 82% and 85%, respectively. The CZE method had an improved separation of charge variants compared with CEX, resulting in the different levels [39]. However, the CZE and CEX showed similar trending for charge variants and the chromatograms are shown in Fig 2.

#### icIEF

**Fig 3:**
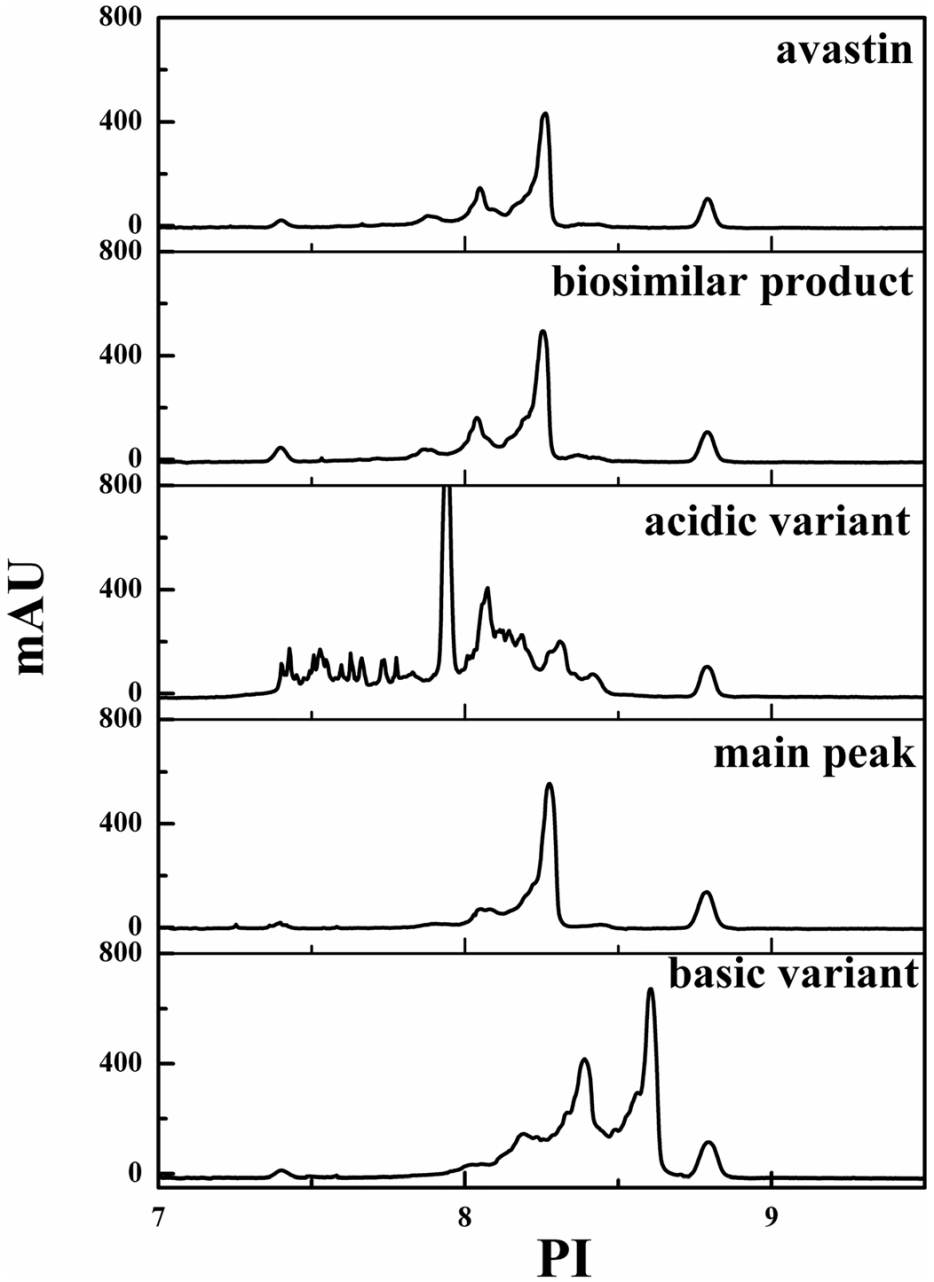
Imaged capillary isoelectric focusing for profile of the isolated charge variants, the biosimilar product and Avastin.

The isoelectric points (pIs) of the isolated charge variants were measured using icIEF [26]. Markers with pI 7.4 and 8.79 were used, and all samples showed pI values between the two markers. The pI values of the biosimilar product and Avastin ranged from 7.8 to 8.4, and the primary peak regions of the acidic variant, basic variant and main peak have a range of 7.9–8.0, 8.3–8.6, 8.2–8.3, respectively. The calculated pI values obtained by icIEF are shown in Fig 3. The results demonstrated that the isolated charge variants had relatively high purity.

#### SEC

**Fig 4:**
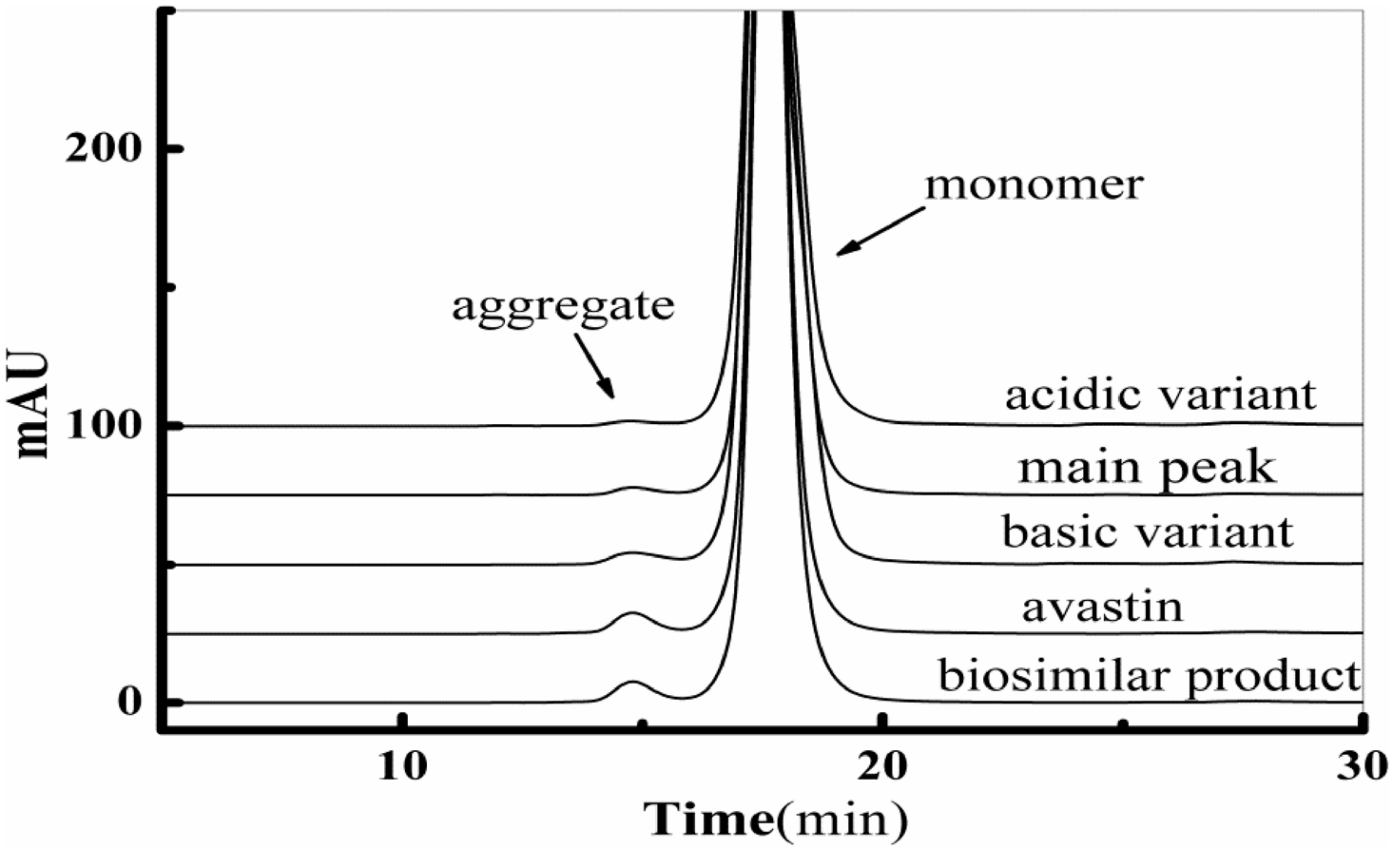
Chromatographic profiles obtained from SEC shown in expanded scale for the isolated charge variant, the biosimilar product and Avastin.

The monomer percentages of the biosimilar product, Avastin, the acidic variant, the basic variant, and the main peak were 98.9%, 98.0%, 99.9%, 98.8% and 99.8%, respectively. The aggregation level of the basic variant appeared to be higher than other charge variants, most likely due to selective enrichment of aggregates in the basic region [11]. The SEC chromatograms are shown in Fig 4.

The CEX-HPLC, CZE, icIEF and SEC results indicated that the isolated charge variants had relatively high purity in terms of charge heterogeneity and size. Therefore, they can be used for further study such as kinetic binding analysis, *in vitro* activity and pharmacokinetics.

### 3.2 Evaluation of the affinity by SPR

Table 2 shows the binding affinity of the samples for Human VEGF165 measured by SPR, revealing no significant difference among the isolated variants, the biosimilar product and Avastin (Student’s t-test, p>0.05).

**Table 2:** Kinetics constants of the isolated charge variants, the biosimilar product and Avastin (mean±standard deviation, n = 3).

Test material	k_a1_(10^5^ M^−1^s^−1^)^a^	k_d1_(10^−5^ s^−1^)^a^	K_D1_(10^−10^ M)^a^	(%CV)^b^
Acidic variant	1.05±0.13	3.12 ±0.25	2.90 ±0.12	4.0
Basic variant	1.39±0.20	3.63 ±0.34	2.64 ±0.13	4.9
Main peak	1.45±0.14	3.02 ±0.45	2.08 ±0.16	7.6
Biosimilar product	1.19±0.14	3.77 ±0.20	3.18 ±0.22	6.9
Avastin	1.27±0.16	4.06±0.23	3.21±0.24	7.3

^a^k_a1_, association rate constant; k_d1_, first dissociation rate constant; K_D1_, dissociation equilibrium constant. All statistical analyses were performed using multiple regressions. Binding of Avastin to Human VEGF165 is shown for reference only.

^b^ CV, coefficient of variation of n = 3

### 3.3 Anti-proliferation potency assay

The results of the anti-proliferation assay show that the relative potency of the acidic variant, the basic variant, the main peak and the biosimilar product against Avastin were 97%, 100%, 103% and 100%, respectively. The acidic variant shows slightly lower potency, and the main peak shows slightly higher potency. The differences in the student’s t test are believed to be within the variability of the assay, suggesting that there is no significant difference *in vitro* activity among the charge variants, the biosimilar product and Avastin (Student’s t-test, p>0.05).

### 3.4 Pharmacokinetics study

**Fig 5:**
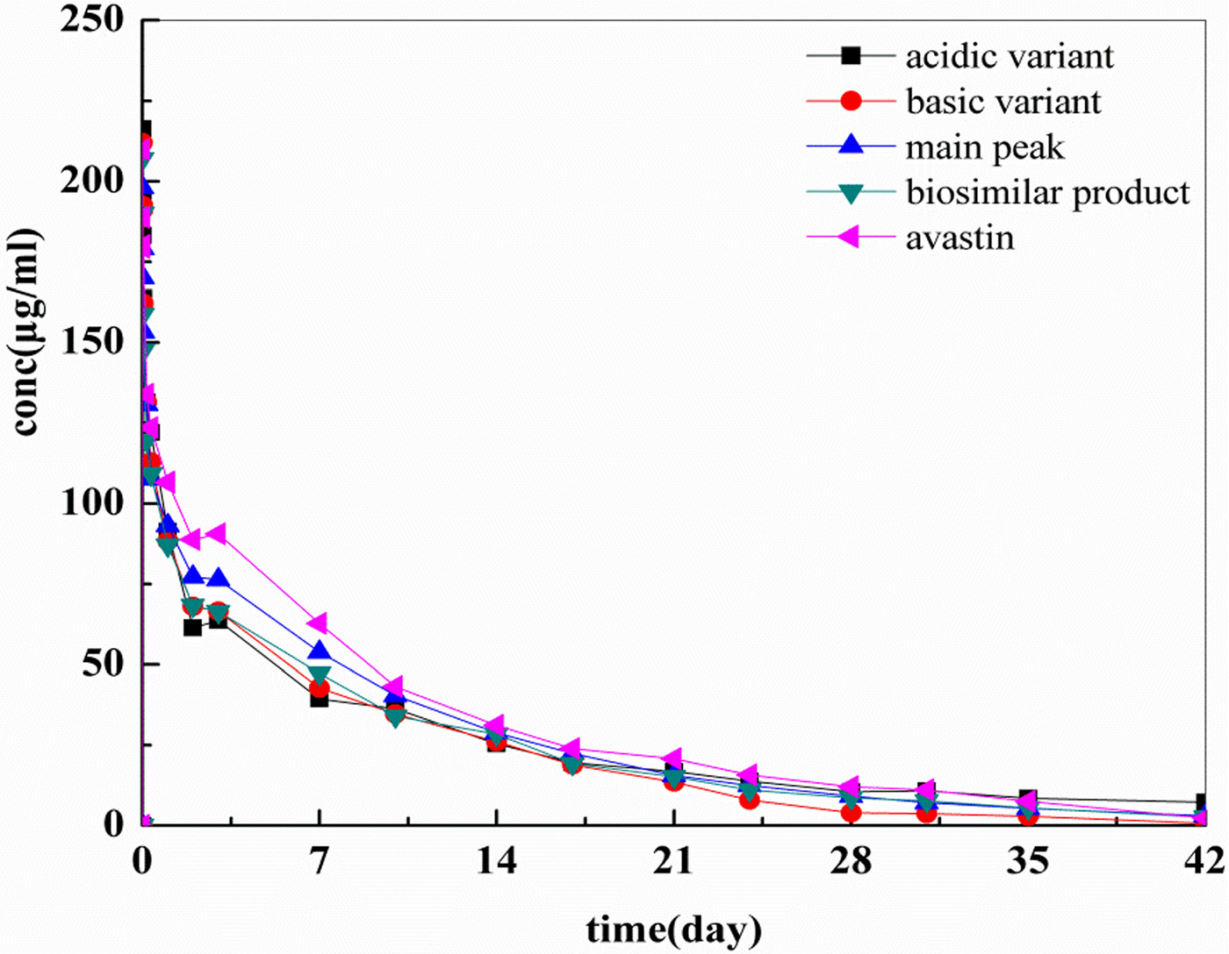
Concentration-time profiles of the isolated charge variants, the biosimilar product and Avastin. The results are shown as the mean ± standard deviation (n = 4 rats/time point).

The serum concentration versus time curves of Avastin, the biosimilar product, and the isolated charge variants are illustrated in Fig 5. The accuracy and reproducibility of the ELISA was examined by determining its intra-assay and inter-assay (within different researchers on different days) on 8 replicates of standards and quality controls (QCs) (correlation coefficients of *R*^2^≥0.999). The intra-assay variation was within 2–10%, while the inter-assay variation was within 4–12% [40–42]. The pharmacokinetics parameters were calculated using non-compartmental analysis (NCA) with Phoenix WinNonlin 6.3. The maximum serum concentration (C_max_) was taken directly from the serum concentration/time profiles, and the corresponding results including C_max_, the terminal elimination half-life (t_1/2_) and the AUC from 0 to 42 days (AUC_0-42d_) are shown in Table 3. No significant difference was observed in t_1/2_ among the isolated charge variants, the biosimilar product and Avastin, which are all approximately 6.85–8.67 days. They also share similar AUC_0-42d_ values (mean ± standard deviation), as the acidic variant, the basic variant, the main peak, the biosimilar product and Avastin have values of 1060.08 ± 256.4, 932.24 ± 108.2, 1130.75 ± 240.3, 1023.29 ± 321.0, 1310.11 ± 394.9, respectively. The comparison of pharmacokinetics parameters was accomplished using SPSS 17.0, and ANOVA showed that there were no differences compared with avastin (P>0.05).

**Table 3:** Serum pharmacokinetics parameters for the isolated charge variants, the biosimilar product and Avastin after a single intravenous injection of 10 mg/kg body weight in rats (n = 4, mean ± standard deviation).

Test material	Study	AUC_0-42d_ (μg*day/mL)	C _max_ (μg /mL)	T_1/2_ (day)
Acidic variant ^b^	IV	1060.08±256.4	216.45±59.07	8.67 ±2.46
Basic variant ^b^	IV	932.24±108.2	212.63±52.30	6.85 ±2.18
Main peak ^b^	IV	1130.75±240.3	202.31±57.06	8.47 ±2.38
biosimilar product ^a^	IV	1023.29±321.0	207.00±41.95	7.68 ±1.75
Avastin	IV	1310.11±394.9	211.97±44.54	8.84 ±1.86

^a^ P>0.05 (biosimilar product vs. Avastin);

^b^ P>0.05 (acidic variant, basic variant, main peak vs. avastin)

## 4. Conclusion

We have demonstrated that the preparation of the acidic variant, the basic variant and the main peak of the Avastin biosimilar can be achieved by the strong cation exchange method with high purity in terms of charge heterogeneity and size distribution. There are no significant differences in pharmacokinetic parameters, which is consistent with previous reports wherein deliberate modification of the pI of an antibody by one unit generated no noticeable differences in the PK [43–44].

Up to now, these data bout avastin and biosimilar have not been reported. In this study, we found that the charge heterogeneity of the biosimilar product exhibited no influence on the in vitro potency and the similar PK profile in rats between charge variant and avastin can be demonstrated. It should be noted that the PK profile of mAb can be affected by various factors, such as glycosylation and aggregation [45]. Thus, charge heterogeneity is simply the tip of the iceberg among the factors that determine product comparability, biological activity and immunogenicity. Therefore, to ensure the product quality, safety and efficacy at all stages of the product life cycle, it is necessary to apply a series of powerful methods to analyze mAbs. This study showed a wider introduction of techniques in antibody characterization and these analytical methods contribute to the biopharmaceutical drug innovation and development. We will make further study to structural evaluation of charge variants using mass spectrometry at top, middle and bottom level, and also attach great importance to the pharmacological activity in malignant cell lines.
